# Trends in task shifting in HIV treatment in Africa: Effectiveness, challenges and acceptability to the health professions

**DOI:** 10.4102/phcfm.v7i1.807

**Published:** 2015-07-30

**Authors:** Talitha Crowley, Pat Mayers

**Affiliations:** 1 Department of Interdisciplinary Health Sciences, Stellenbosch University, South Africa; 2 Department of Health and Rehabilitation Sciences, University of Cape Town, South Africa

## Abstract

**Background:**

Task shifting has been suggested to meet the demand for initiating and managing more patients on antiretroviral therapy. Although the idea of task shifting is not new, it acquires new relevance in the context of current healthcare delivery.

**Aim:**

To appraise current trends in task shifting related to HIV treatment programmes in order to evaluate evidence related to the effectiveness of this strategy in addressing human resource constraints and improving patient outcomes, challenges identified in practice and the acceptability of this strategy to the health professions.

**Method:**

Electronic databases were searched for studies published in English between January 2009 and December 2014. Keywords such as ‘task shifting’, ‘HIV treatment’, ‘human resources’ and ‘health professions’ were used.

**Results:**

Evidence suggests that task shifting is an effective strategy for addressing human resource constraints in healthcare systems in many countries and provides a cost-effective approach without compromising patient outcomes. Challenges include inadequate supervision support and mentoring, absent regulatory frameworks, a lack of general health system strengthening and the need for monitoring and evaluation. The strategy generally seems to be accepted by the health professions although several arguments against task shifting as a long-term approach have been raised.

**Conclusion:**

Task shifting occurs in many settings other than HIV treatment programmes and is viewed as a key strategy for governing human resources for healthcare. It may be an opportune time to review current task shifting recommendations to include a wider range of programmes and incorporate initiatives to address current challenges.

## Abstract (fr)

**Tendances dans le transfert de tâches dans le traitement du VIH en Afrique : efficacité, défis et acceptabilité relatifs aux professions de la santé.**

**Contexte:**

On a suggéré que le transfert de tâches pouvait répondre à la demande en matière de début de traitement et de prise en charge de davantage de patients sous traitement antirétroviral. Bien que l'idée du transfert des tâches ne soit pas nouvelle, elle acquiert une nouvelle importance dans le contexte de la prestation de soins de santé actuelle.

**But:**

Estimer les tendances actuelles en matière de transfert des tâches lié à des programmes de traitement du VIH afin d’évaluer les preuves de l'efficacité de cette stratégie dans le traitement des contraintes en termes de ressources humaines et dans l'amélioration des résultats des patients, de déterminer les défis identifiés dans la pratique et le caractère acceptable de cette stratégie pour les professions de la santé.

**Méthode:**

Des bases de données électroniques ont été recherchées pour trouver des études publiées en anglais entre janvier 2009 et décembre 2014. Des mots-clés tels que « transfert des tâches », « traitement contre le VIH », « ressources humaines » et « professions de la santé » ont été utilisés.

**Résultats:**

Les preuves suggèrent que le transfert de tâches est une stratégie efficace pour faire face aux contraintes en termes de ressources humaines au sein des systèmes de soins de santé dans de nombreux pays et fournit une approche peu coûteuse sans compromettre les résultats des patients. Les défis incluent un soutien en termes de supervision et un mentorat insuffisants, l'absence de cadres réglementaires, un manque de renforcement général du système de santé et la nécessité d'un suivi et d'une évaluation. La stratégie semble être généralement acceptée par les professions de la santé, bien que plusieurs arguments allant à l'encontre du transfert de tâches en tant qu'approche à long terme aient été émis.

**Conclusion:**

Le transfert de tâches se produit dans de nombreux contextes autres que les programmes de traitement du VIH et est considéré comme une stratégie clé pour gérer les ressources humaines dans le domaine de la santé. Il est peut-être aujourd'hui opportun d'examiner les recommandations actuelles en termes de transfert de tâche pour y inclure un plus large éventail de programmes et intégrer les initiatives visant à relever les défis actuels.

## Introduction

Globally the number of people living with HIV is increasing as more people are accessing life-saving antiretroviral therapy (ART), and the world is within reaching the goal of providing ART to 15 million people by 2015.^1^ However, in recent years there has been a crippling shortage in well-trained healthcare workers worldwide. At least 57 countries are affected, of which 36 countries are in sub-Saharan Africa.^2^ The reasons for this disparity in qualified staffing in health services in Africa include a lack of training capacity, inefficient deployment of health workers, poor remuneration and limited budgets allocated to health services.^2,3^

In 2006, the World Health Organization (WHO) recognised that decreased access to care is associated with health worker shortages, especially in sub-Saharan African countries. Task shifting was then accepted globally as a strategy to improve access to care and meet the demand for initiating and managing more patients on ART.^4,5^ Consolidated guidelines proposed by the WHO in 2013 have advocated the goals of keeping HIV-infected people healthy and reducing HIV transmission, thus emphasising the importance of equitable access to ART.^6^ The Fast-Track strategy of the United Nations’ Joint Programme on HIV/AIDS (UNAIDS) calls for a rapid scale-up of HIV prevention and treatment strategies in order to achieve the goal of 95% of people living with HIV knowing their status, 95% ART coverage and 95% virologic suppression rates by 20.30.^7^ This necessitates a further scale-up of the provision of ART, whilst making sure that patients are retained in care.^8^ Decentralisation and task shifting have been referred to as the two pillars of the public health approach to scale up ART in resource-limited settings.^9^

Task shifting refers to transferring tasks to healthcare workers who have not conventionally performed these tasks as part of their scope of practice.^10,11^ These workers are more readily available, completed shorter training and have fewer qualifications.^12^ Clearly outlined tasks are delegated to healthcare workers who have received specific skills-based training, and this approach may extend to persons specifically trained to perform a limited task only.^13^ The aim is to make efficient use of human resources for health in order to minimise delays in health service delivery without compromising the standard of care,^10^ with the particular aim of improving the health of extremely vulnerable populations.^13^

**FIGURE 1:**
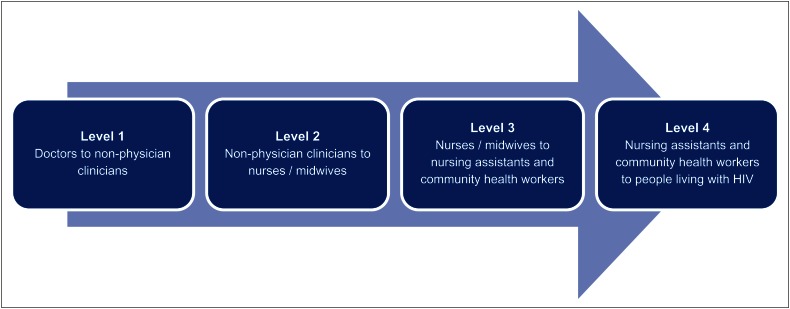
The process of task shifting as adapted from the World Health Organization global recommendations and guidelines.^10^

Task shifting is also referred to as the rational distribution of tasks amongst members within health workforce teams.^10^ The four levels of task shifting (Figure 1), in which health workers of the first three levels take on tasks of senior cadres, are based on the extension of the scope of practice of:

- non-physician clinicians (NPCs)
- nurses and midwives
- lay health workers or community workers
- and people living with HIV to self-manage aspects of their care.^10^

In this article, task shifting refers primarily to the shifting of tasks from doctors to NPCs or nurses (levels 1 and 2) and the further shift of tasks to lay health workers (level 3). NPCs are a cadre of health workers who are not trained as doctors, but take on many diagnostic and clinical functions of doctors.^14^ NPCs, also known as health officers, clinical officers, physician assistants, nurse practitioners or nurse clinicians, have been identified as part of the health workforce in 25 countries in sub-Saharan Africa.^14^ Lay health workers have no professional or paraprofessional qualifications and perform context-specific healthcare functions for which they received informal training.^15^

The word ‘substitution’ has been used when referring to task shifting, especially when referring to nurses taking on the role of doctors.^16^ Words such as ‘task delegation’ or ‘task sharing’ imply that the person delegating or sharing the task remains responsible for the final outcome.^17^ The WHO made several recommendations for the implementation of task shifting in the context of HIV services, such as the development of nationally endorsed frameworks to support the strategy, creating an enabling regulatory environment, measures to ensure quality care, maintaining stability and reorganisation of clinical care.^10^

The aim of this paper is to appraise the current trends in task shifting of HIV treatment and care to lower cadres of healthcare staff to evaluate evidence related to the effectiveness of this strategy in addressing human resource constraints and improving patient outcomes, challenges identified in practice and the acceptability of the strategy to the health professions. Attention is also drawn to the recommendations of the WHO and how countries have implemented these.

## Methods

The electronic databases PubMed, CINAHL and Google Scholar were searched to identify relevant papers published between January 2009, following the publication of the WHO task shifting recommendations, and December 2014 for review. Medical subject heading (MeSH) terms included ‘task shifting’, ‘HIV treatment’, ‘human resources’ and ‘healthcare professions’. Quantitative and qualitative studies published in English were included if they presented evidence on task shifting. We identified 395 articles of which the titles and abstracts were subsequently screened for appropriateness. The full texts of 67 articles, of which eight were systematic reviews, were assessed and 46 articles were included in this review. The majority of the reported research was conducted in Africa and sub-Saharan Africa. A critical analytical approach draws attention to current trends in task shifting and evidence related to the successes as well as the challenges of the strategy.

## Review findings

### Trends in task shifting: Towards community-supported care

Many countries, especially in sub-Saharan Africa, have adopted task shifting as an inevitable approach to alleviate human resource constraints and expand HIV programmes.^16,18^ Task shifting seems to include more than only HIV services and is practised in almost all health facilities, either formally or informally, across a number of disciplines, including midwifery, surgery and non-communicable disease management.^3,8,19,20,21,22,23^ The extent to which task shifting is implemented seems to depend on the specific discipline or service, health system capacity and health needs. In the context of HIV services, the most often reported levels of task shifting are from doctors to NPCs, nurses or midwives, and the shifting of tasks such as phlebotomy, dispensing and counselling from nurses or midwives to lay health workers.^18,24^

Lay health workers are used increasingly, especially in the context of disease prevention, health promotion, treatment support, counselling and home-based care.^2^ A systematic review on the roles of community health workers in HIV care in sub-Saharan Africa reported that this group of health workers were instrumental in promoting the quality of HIV services and that their presence in clinics reduced waiting times for patients and the workload of other health workers.^24^ As most community health workers live in the communities they serve, challenges of cultural and language competence are mitigated.^25^

In South Africa, a large infrastructure of lay health workers has developed in response to the HIV epidemic. In 2004, the estimated number of these workers (40 000) was almost equivalent to the number of professional nurses (43 660) working in the public sector.^26^ Incorporating such an extensive lay health worker infrastructure is in line with the country's strategy for the re-engineering of primary healthcare and steps have been taken to design a formal qualification for such health workers.^27^ In Malawi, lay health workers (also known as health surveillance assistants) comprise 30% of the health workforce.^28^ In Malawi and Kenya, lay health workers perform mostly health prevention and promotion tasks in communities, but are increasingly performing curative tasks.^28,29^

Owing to the increasing HIV treatment burden, care has gradually moved from hospitals to decentralised healthcare facilities and has now further expanded to community-supported care for stable patients.^12,30^ In Kenya, lay workers’ community-based care for stable patients, supported by mobile technology, reduced clinic visits without compromising patient outcomes.^31^ Other successful community-based interventions, such as adherence clubs, have also been reported.^30,32^

The task-shifting process for HIV treatment and care is well described in most contexts of care, but it is evident that it may not be the same for other, non-HIV services.^19^ This may be as a result of the WHO recommendations focusing specifically on task shifting in the context of HIV. Although the potential for wider application is recognised, detailed guidelines for application to other services are not provided.

### Effectiveness of task shifting in addressing human resource constraints and improving patient outcomes

The expansion of ART programmes and improved access to care in the midst of pervasive human and other resource constraints have been attributed to liberal task shifting strategies.^18,33^ In most settings, task shifting has been implemented without significant increases in human resources.^4,18^ The effect of task shifting has been evaluated in various countries, including South Africa, Ethiopia, Kenya, Malawi, Lesotho, Botswana, Swaziland, Nigeria, Cameroon, Zambia, Uganda, Mozambique and Rwanda.^18,34^ A systematic review of the effect of task shifting found that the strategy can offer high-quality, cost-effective care to more clients than a physician-centred model.^4^ The strategy also appears to be acceptable to patients.^34^

With regard to HIV treatment, care provided by non-physicians (including NPCs, professional nurses and midwives) is comparable to physician-provided care,^35^ may reduce loss-to-follow-up rates and improve patient satisfaction.^36^ Reduced loss to follow-up may also be attributed to decentralisation associated with task shifting as patients are receiving care closer to their homes.^12^ However, neither decentralisation nor task shifting is a magic bullet for improving patient outcomes: a South African study reported that loss to follow-up is challenging whether care is decentralised or not and that rates are increasing with each calendar year since a patient started ART.^37^

Models based on decentralised HIV care do not compromise the health outcomes of patients, even when the patients are treated in the community rather than at primary care health facilities and hospitals.^12^ It is also argued that task shifting may improve quality of care, as tasks such as patient education may be better delivered by nurses or lay health workers.^16,24,26^ It may even lead to better retention in the healthcare workforce, especially in remote areas, by providing career opportunities for lower cadres of staff.^16,19^

Current research therefore supports the trend of adopting task shifting for HIV treatment and advocates for the expansion of task shifting policies.^24,36^ However, many studies included in recent systematic reviews^12,34^ were in the context of a range of support structures and investments to ensure delivery, including training and supervision. In practice, task shifting may be complex and be implemented in dissimilar forms, with substantial variation in the cadre, training and competency levels of health workers to whom tasks are shifted, for example NPCs or nurses. This makes comparisons of the success of the strategy in different countries problematic.^16,18^ The generalisation of findings may not necessarily extend to wider healthcare systems and is dependent on the setting and organisation of care.^16,34,36^ However, it is evident that task shifting has improved access to care for people who may not have had any access if the strategy had not been implemented. Fulton et al.^20^ argue that studies should actually compare the outcomes of task shifting models with that of standard care, as many patients would not have received any treatment if not for task shifting.

### Challenges identified in practice

Task shifting is not the remedy for a dysfunctional health system. For task shifting to succeed, systems and human resource support is required.^4^ Although task shifting can improve access to and utilisation of services, costs of care do not necessarily decrease, as evidenced by a study in Ethiopia which reported that task shifting has not resulted in a significant decrease in the cost of ART care.^38^ In South Africa, nurse-led ART has been associated with higher health service costs,^39^ which may be attributed to the cost of supporting nurses through training and mentoring, limiting the potential savings.^34,39^

It is more costly to train and employ doctors and professional nurses than lay health workers, yet more skilled and experienced healthcare workers have been associated with better patient outcomes, which may partially offset cost of employing better-qualified staff.^40^ Conversely, some countries may not be able to afford to employ a large number of well-trained qualified staff.^3^ Task shifting is therefore a viable option for efficiently providing services that may otherwise not have been available.^19,20^ Lehmann et al.,^5^ and more recently also Lanktree et al.,^41^ have argued that the long-term success of task shifting hinges on political and financial commitment, reorganisation of health teams, clear scopes of practice, recognition, simplified guidelines, regulatory frameworks and training, support and monitoring of infrastructure.

Several challenges to the implementation of task shifting according to WHO guidelines and recommendations have been reported. These challenges are discussed briefly.

### Support, training and care reorganisation

Task shifting is complex and can be successful only when accompanied by training, reorganisation of services, mentoring, supervision and ongoing support from existing health system structures.^4,18,35^ If such a strategy is implemented without sufficient clinical and managerial support, it could be less effective than the outcomes observed in clinical trials.^31,34^

When implemented in an already burdened and dysfunctional health system, task shifting alone is not enough to improve efficiency and ensure quality of care.^18^ It may lead to the depletion of already limited health resources.^36^ Patient outcomes may be compromised in settings where nurses are already overworked if current tasks of nurses are not shifted to lower levels of workers.^36,42^ In South Africa, a high percentage of professional nursing positions remains vacant and the reality that task shifting relies heavily on nurses to take on new tasks and to supervise other, non-professional cadres of staff is therefore problematic.^11^

Adequate knowledge and skills are required for supportive supervision, but health workers capable of providing supportive supervision are also experiencing heavy workloads. Consequently, tasks may be shifted without the guarantee of supportive supervision.^3,19,28,43^ Staff training is central to the implementation of task shifting, but a major limitation to this is high staff turnover.^44^ Measures to limit staff turnover and increase mentoring and supervision capacity therefore need to be planned from the outset.^4^

A lack of health system resources and support, inadequate infrastructure and equipment, ineffective guidance from managers, increasing workloads and unresolved remuneration issues have been identified as barriers to the implementation and efficiency of task shifting.^3,23,43^ Other barriers identified include a shortage of nurses working at primary care facilities, ongoing training in clinical skills and the expansion of clinical mentoring.^45^ A number of authors have reported that there is no structured system in place or resources for supervision and mentoring after training.^19,36,42,46^

Assefa et al.^9^ reported that the scale-up of ART in Ethiopia has been made possible through a range of interventions alongside task shifting. Health system strengthening, community mobilisation, case management support and patient information systems were reported as key contributory initiatives to effective scale-up. The implementation of a task shifting strategy in Malawi required regular and sustained peer-based support for health workers and their mentors. Linking training to continuous professional development credits further strengthened training uptake.^44^

Currently, there is still limited evidence about the key barriers and enablers to effect successful implementation of task shifting in resource-constrained settings with weak health systems and inadequate supervisory support.^16^ Most studies to date have focused on settings with vertical programmes, which means there is minimal evidence to inform decision makers about task shifting in special risk groups and integrated health system programmes. In their systematic review, Kredo et al.^34^ found moderate-quality evidence that substituting nurses for doctors to initiate and monitor ART in adults will not compromise quality of care, but that there were no studies to inform practice in special risk groups. Two examples for which there is no clear evidence are whether nurses should be allowed to prescribe both ART and other chronic medication to patients with co-morbid conditions and whether midwives can prescribe ART in the context of antenatal care. In Malawi, vertical disease-specific programmes were reported to lead to competing demands being placed on healthcare workers, which can cause role confusion and hamper the effectiveness of task shifting.^28^ A recent systematic review of health system barriers and enablers for ART delivery during pregnancy and post partum identified gaps between antenatal care and HIV services that resulted in significant dropout along the maternal ART cascade. The authors suggested that this situation may be improved if tasks such as antenatal care and the provision of ART are integrated.^47^ Although there is a drive towards increasing service integration, it is not clear what the ideal balance between depth and breadth of shifted tasks should be to ensure quality and efficiency.

### Competency assessment and quality assurance mechanisms

A three-pronged approach to task shifting, which comprises training, on-site clinical mentoring and continuous quality assurance, has been advocated.^2,48^ The WHO recommends that countries implementing task shifting should ensure that the performance of all cadres of health workers can be assessed against clearly defined roles, competency levels and standards.^10^ Morris et al.^48^ describe a comprehensive, continuous quality assurance programme for task shifting that was implemented in Zambia through (1) evaluation of clinical care via targeted chart reviews and monthly site reports from electronic medical records, (2) feedback and training in areas of poor site performance, and (3) an exchange programme between clinics to improve overall clinical quality. No other published studies describing specific quality assurance or clinical governance mechanisms for the wider implementation of task shifting could be found; however, routine data from a study in Khayelitsha, South Africa, showed an improvement in the quality of ART management after clinical mentoring.^49^

Some studies from South Africa, Tanzania, Uganda, Mozambique and Zambia have reported that tasks were delegated without prior training and that a ‘trial-and-error’ approach was followed.^3,19,23,42^ A survey of 15 African countries (including the aforementioned as well as other countries) indicated that the majority of countries in which task shifting of ART was allocated to nurses follow an in-service training approach; however, such training was generally not accredited or approved by national nursing and midwifery councils.^50^ In addition, there is no evidence that competencies required by task shifting are being introduced in pre-service education.^8,50^

It is also costly to develop new training programmes^36^ and clarity with regard to whether training should be provided by higher education institutions or private, 'informal’ training institutions is needed. Informal training may be problematic because of a lack of standardisation within and across training programmes and countries, and the quality may also be questionable.^50^ In Malawi, an in-service training programme for NPCs was suspended owing to observational data indicating only 10.6% of clinical encounters were correctly managed after training.^51^ In a South African study, only 60% of nurses were able to pass an open-book test after didactic in-service training, yet 62% of nurses who had failed the test also reported to initiate patients in practice following the training.^45^

### Regulatory and accreditation systems

The WHO recommends accreditation and certification programmes for newly trained health workers in order to provide career paths, as well as recognition of prior learning for those who have already taken on tasks outside their scope of practice through pragmatic responses to healthcare needs.^10^ Most countries use documents from their ministry of health, such as ART guidelines, to authorise ART prescription without including the long-term WHO-recommended processes such as credentialling and regulation.^50^ Many nursing and midwifery councils in eastern, central and southern Africa do not have the resources or capacity to undertake changes associated with practice regulation.^52^

In South Africa, there is no formal regulatory system for nurses to prescribe ART^53^ or for the scope of practice of lay health workers. Although it is reported that section 56(6) of the South *African Nursing Act* (33 of 2005) allows for nurses to be authorised to prescribe ART,^17^ nurses are concerned about the lack of certification from the South African Nursing Council.^43^ Although task shifting is permissible in many countries, there is no legal protection for health workers if they act outside of their scope of practice.^2,3^ The scope of practice of lay health workers is constantly being expanded to enable wider service delivery. This sometimes includes curative services and tasks not in their job description.^28,29^ As these health workers are unregulated, there is no control over their training or the quality of their work.^24,40^ It has therefore been advocated that their training be appropriately accredited, certified and recognised.^24,29^ Roles and associated competency levels should be clearly defined for existing health workers as well as for those cadres newly created by the task-shifting approach.^17^

Formal career pathways for lower cadres that have an extended scope of practice seem to be absent in most countries. Nurses and lay workers are taking on additional tasks for which they are not adequately remunerated^24,36^ and no incentives or career promotion frameworks exist.^3,19,23,24,26^ Investment in policy, regulation and pre-service education is needed to ensure sustainable task-shifting interventions.^8,24,50,52^

### Acceptability of task shifting to the health professions

Social dynamics in healthcare teams may be affected by task shifting policies.^4^ Issues such as professional boundaries, dominance and power have been identified.^11^

Professional nurses seem to embrace task shifting and feel empowered by their expanded roles.^42^ They accept task shifting in relation to training and the acquisition of knowledge and skills.^53,54^ Nurses have also expressed feelings of emotional reward, accomplishment, prestige and improved morale.^55^ A South African study by Davies et al.^42^ found that nurses experienced emotional reward through building relationships with patients and that they feel empowered by the realisation that they can also act as mentors to other nurses. They are willing and receptive to the idea as it is in the best interest of the patients, yet they acknowledge that circumstances are sometimes less than ideal.^42^ The need for clear role definitions of different providers and stratification of roles at primary-care level to make task shifting more effective with respect to quality patient care and resource utilisation has been highlighted.^43^

Although task shifting seems to be generally accepted as a necessary strategy to increase access to care, there are conflicting opinions amongst doctors, in particular with respect to the perceived competency of nurses to manage HIV-infected persons.^55^ A Ugandan study found that task shifting was unacceptable to most health managers and policy makers, as they considered the lower categories of health workers to be incompetent and overworked, which is exacerbated by inadequate support and supervision.^3^ In Mozambique and Zambia, there has been concern that task shifting carries risks for patients, staff and management, although the concern was related mostly to insufficient support systems and workload constraints.^23^ Task shifting in Tanzania was considered by respondents to be a short-term solution whilst the government worked towards more sustainable ways of addressing the health personnel crisis.^19^ Lay health workers in Malawi reported confusion about their ever-expanding job description, but were willing to expand their scope of practice if appropriate training and resources were provided.^28^

Professional cadres of health workers can view task shifting either as alleviating their burden of tasks and improving access to care or as encroaching upon their profession and compromising the quality of care. As health professionals are accountable for their own practice, Scott et al.^56^ argue that ‘accountability implies autonomy and choice’ and if there is no alternative to inadequate care, choice does not exist. For the health service to demand an extended role of nurses and other health workers, the necessary resources and an enabling environment that facilitates such accountability must be ensured.^56^

The categories of healthcare workers have become increasingly diverse over the last 20 years. This has been particularly noticeable as a result of the increased need to access chronic care and ART. The diversity of the healthcare workforce created by new cadres of health workers may give rise to complex intra- and interprofessional boundary disputes.^11^ This strategy may be seen by some as de-professionalising the workforce through a loss of authority over certain aspects of work.^11^ Some instances of professional protectionism have emerged, such as doctors arguing that nurses have insufficient training and skills to adequately perform tasks that were formerly only in the ambit of the medical profession, and nurses feeling that lay health workers are invading their profession. As a result, lower cadres of health workers may not feel comfortable in embracing task shifting.^2^

In South Africa, nurses were resistant to lay health workers wearing uniforms, in an attempt to maintain professional boundaries.^26^ Nurses reported that they did not supervise lay health workers, despite working closely together. Some district-level informants in Tanzania were not supportive of legalising task shifting, as they feared professional conflicts between workers of different cadres.^19^ A South African study, however, reported that health workers functioned well in a supportive multidisciplinary team and that the changing nature of HIV services was the driver of a team approach to care.^43^

Although the effect of role changes on professional status is still unclear, professional labels associated with a particular service, ownership of a body of knowledge, autonomy and authority remain uncontested. Lower levels of workers are unlikely to be afforded appropriate recognition and status, even if they are performing the tasks of higher-level workers.^11^

Several sound arguments both for and against the long-term implementation of task shifting have been extrapolated, categorised and presented in Table 1. Pragmatists consider the issue in a logical, practical way, arguing that task shifting is inevitable in the context of the broader health system and that the system and professionals should adapt accordingly. In contrast, purists value quality of care and the status of the health professions.

**TABLE 1:** Contrasting arguments for and against task shifting.

Pragmatic	Purist
Rational distribution of tasks in care teams. New cadres perform additional tasks (e.g. defaulter tracing), which will improve the quality of patient care.	Delegation of tasks to already overburdened health workers.
Expanded roles lead to empowerment.	Lowering the required level of competence promotes deprofessionalisation.
Job creation and career progression.	No job description, performance framework or increase in remuneration.
Increased community access to basic health care and improved community engagement. Efficiency of services improves patient satisfaction.	A decrease in quality of care; infringement of the rights of the community to receive care from skilled health workers.
Increased cost effectiveness (increasing the number of services provided at a given quality and cost). Alleviate skill-mix imbalances.	Decisions are based on economic and budget constraints rather than on actual health worker shortages; hidden costs associated with training and supervision.
Only a basic level of training, focused on specific skills, is needed. Some tasks require only focused training.	Comprehensive care requires advanced education, professionalism and ethics. Roles and tasks are continually evolving, requiring ongoing training.
A task-oriented approach improves efficiency of care.	A task-oriented approach causes fragmentation in care.
Ethical responsibility to adapt to the needs of the community and provide equitable access to healthcare.	Ethical responsibility to protect the community and healthcare workers from harm.

## Implications and recommendations

Task shifting has been widely adopted and seems to be an effective strategy for providing services that may otherwise not be available.^20^ Several challenges to the implementation of task shifting have been reported. The strategy generally seems to be well accepted by the various health professions although arguments against the strategy as a long-term approach to care have been raised.

In its current form, task shifting should not be considered as a remedy for current healthcare delivery problems. Efforts to seek long-term sustainable solutions should continue.^19,36^ For most developing countries, however, task shifting is a reality in healthcare service delivery and is already moving beyond the clinic to community level. It is not likely to be a short-term measure and is seen by Margret Chan, director-general of the WHO, as an approach that offers long-term potential for all primary healthcare services.^10^ Task shifting initiatives should therefore be flexible and adaptable to a specific context and ground-level needs. Research on various models of task shifting strategies in different contexts and the impact on patient outcomes should continue.

If doctors’ tasks are shifted to NPCs and nurses, these latter groups’ tasks should, in turn, shift to other non-professional cadres.^36^ The re-engineering of primary healthcare strategies relies heavily on the use of lay health workers in communities, supervised by nurses.^11^ Nurses will become increasingly responsible for overseeing the tasks of lay health workers. Tasks of lay health workers are continually expanding yet their roles are not clearly defined, and as such this cadre remains unregulated in many countries and workers experience limited regulatory protection.^3,15,24,57^ Furthermore, it is not clear how supportive supervision should be defined in community-based care settings. As lay health workers take on caring roles and function autonomously, especially in rural settings, they should be appropriately trained and have clear role descriptions.

Evidence indicates that the implementation of task shifting according to WHO guidelines and recommendations is problematic, especially in resource-constrained countries. The complexity and context sensitivity of the approach make a standardised method of implementation impossible. Implementing task shifting in isolation is unlikely to address long-term sustainability of programmes. Further research is needed with regard to the interventions necessary for adequate support of personnel and effectiveness of services. Task shifting may need to form part of an interdisciplinary team approach, with patient self-management as a central focus.

Key stakeholders need to engage in discussions about whether task shifting should be programme specific or general. Programme-specific task shifting strategies may become inefficient in the near future, especially in the African context with its diverse needs and high co-morbidity rates. This is illustrated by evidence indicating that the tasks of community health workers are continually expanding. There is, however, still insufficient evidence to support task shifting for integrated care. Countries need to look beyond the immediate ‘crisis management’ approach to task shifting and focus on longer-term strategies that include enhanced training and monitoring of infrastructure. New models for supportive supervision, with expert clinicians as team leaders, are needed to support task shifting and ensure quality of care. Other initiatives, such those based on mobile technology, may be used to provide support to nurses and lay health workers in resource-limited settings.^31,57^

With the increasing number of patients receiving chronic treatment, triage models, in which stable patients are managed in community adherence clubs, are needed to increase service efficiency.^30,32^ Public-private partnerships may be another way to share patient loads as well as clinician expertise.

## Limitations

This limited review included only studies published in English since 2009, with the aim of informing benefits and challenges of the implementation of task shifting in the African context.

## Conclusion

To date, research indicates that task shifting is effective in providing potentially life-saving treatment and care to a greater number of clients. It is a key strategy for managing human resources for health. A lack of implementation may therefore be considered unethical and implementation without adequate health system strengthening could lead to failure. Although task shifting has been adopted by many countries and has been implemented in many diverse settings, there is a lack of fidelity to WHO guidelines, especially concerning regulatory frameworks, supportive supervision, monitoring, and evaluation systems. Leadership and commitment from governing structures are essential to address these concerns and provide solutions. The participation of professional regulatory bodies in decision-making processes regarding task shifting is essential to ensure future success.

It has been seven years since the WHO initially introduced recommendations on task shifting. Although these recommendations did not exclude the possibility of wider application, guidelines were specific to the context of HIV-related services. It may now be an opportune time to review and modify the WHO recommendations to include a wider range of programmes and incorporate initiatives to address current challenges.
